# Regimen-dependent synergism and antagonism of treprostinil and vildagliptin in hematopoietic cell transplantation

**DOI:** 10.1007/s00109-019-01869-8

**Published:** 2019-12-24

**Authors:** Eva Zebedin-Brandl, Madeleine Themanns, Zahra Kazemi, Shahrooz Nasrollahi-Shirazi, Marion Mussbacher, Elizabeth Heyes, Katrin Meissl, Michaela Prchal-Murphy, Wolfgang Strohmaier, Guenther Krumpl, Michael Freissmuth

**Affiliations:** 1 Institute of Pharmacology and the Gaston H. Glock Research Laboratories for Exploratory Drug Development, Medical University of Vienna, 1090 Vienna, Austria; 2 Institute of Vascular Biology, Centre of Physiology and Pharmacology, Medical University of Vienna, 1090 Vienna, Austria; 3 Institute for Medical Biochemistry, University of Veterinary Medicine, 1210 Vienna, Austria; 4 Institute of Animal Breeding and Genetics, University of Veterinary Medicine, 1210 Vienna, Austria; 5 Institute of Pharmacology and Toxicology, Department of Biomedical Sciences, University of Veterinary Medicine, 1210 Vienna, Austria; 6 SciPharm SàRL, L-6689 Mertert, Luxembourg; 7 MRN Medical Research GmbH, Postgasse 11, 1010 Vienna, Austria

**Keywords:** Migration, Homing, Engraftment, Hematopoietic cell transplantation/HCT, DPP4/CD26-inhibitor, Prostanoid receptor agonist, Drug combination therapy

## Abstract

**Abstract:**

The cell dose in umbilical cord blood units is a major determinant for the outcome of hematopoietic cell transplantation. Prostaglandin analogs and dipeptidylpeptidase-4 (DPP4/CD26)-inhibitors enhance the ability of hematopoietic stem cells (HSCs) to reconstitute hematopoiesis. Here we explored the synergism between treprostinil, a stable prostaglandin agonist, and the DPP4/CD26-inhibitor vildagliptin. The combination of treprostinil and forskolin caused a modest but statistically significant increase in the surface levels of DPP4/CD26 on hematopoietic stem and progenitor cells (HSPCs) derived from murine bone and human cord blood. Their migration towards stromal cell-derived factor-1 (SDF-1/CXCL12) was enhanced, if they were pretreated with treprostinil and forskolin, and further augmented by vildagliptin. Administration of vildagliptin rescued 25% of lethally irradiated recipient mice injected with a limiting number of untreated HSPCs, but 90 to 100% of recipients injected with HSPCs preincubated with treprostinil and forskolin. The efficacy of vildagliptin surpassed that of treprostinil (60% rescue). Surprisingly, concomitant administration of vildagliptin and treprostinil resulted in poor survival of recipients indicating mutual antagonism, which was recapitulated when homing of and colony formation by HSPCs were assessed. These observations of regimen-dependent synergism and antagonism of treprostinil and vildagliptin are of translational relevance for the design of clinical trials.

**Key messages:**

- Pretreatment with treprostinil increases surface levels of DPP4/CD26 in HSPCs.
- Vildagliptin enhances in vitro migration of pretreated HSPCs.
- Vildagliptin enhances in vivo homing and engraftment of pretreated HSPCs.
- Unexpected mutual antagonism in vivo by concomitant administration of vildagliptin and treprostinil.

**Electronic supplementary material:**

The online version of this article (10.1007/s00109-019-01869-8) contains supplementary material, which is available to authorized users.

## Introduction

The merit of hematopoietic cell transplantation (HCT) is undisputed [1]. Umbilical cord blood (CB) is an easily accessible source of hematopoietic stem and progenitor cells (HSPCs) for HCT [2]. The small number of HSPCs in single units of umbilical CB preparations is a major limitation: depending on the extent of immunological mismatch, the dose of nucleated cells must exceed 2.5 × 10^7^/kg to 5 × 10^7^/kg to achieve 50% 1-year overall survival after myeloablative transplantation [3]. This cell dose cannot be readily achieved with single CB units. Accordingly, it is of interest to find means for stimulating engraftment of hematopoietic stem cells (HSCs). Translation is facilitated, if this stimulation can be achieved by drugs already approved for clinical use in other indications: their safety profile in people is well understood. Raising cAMP by activation of Gαs enhances engraftment of HSCs [4]. This can be achieved by pretreating HSCs in vitro with prostanoid E and I receptor agonists, e.g., dimethyl-PGE2 (dmPGE2) [5, 6] or treprostinil [7]. Treprostinil has two advantages: it is an approved drug and it can also be administered in vivo*,* which further enhances bone marrow reconstitution in recipient animals [7]. Similarly, genetic deletion or inhibition of dipeptidyl peptidase-4 (DPP4/CD26) promotes the reconstitution of the bone marrow after HCT [8, 9]. The beneficial action of treprostinil in HCT is accounted for by an increase in the expression of CXCR4 [7]. Stromal cell-derived factor-1 (SDF-1/CXCL12), the cognate ligand of CXCR4, is a chemoattractant for HSPCs [10] and is degraded by DPP4/CD26 [11].

Accordingly, we explored the hypothesis that the outcome of HCT can be further improved by combining two approved drugs, i.e., treprostinil and vildagliptin. Our experiments define the conditions, under which this improvement can be achieved. We show that recipient mice benefitted most from a sequential regimen, in which HSPCs were first incubated in the presence of treprostinil and forskolin and the recipient animals subsequently treated with vildagliptin. This regimen was superior to a schedule, where the recipient animals were also administered treprostinil in vivo. In contrast, concomitant administration of vildagliptin and treprostinil to recipient animals resulted in mutual antagonism.

## Materials and methods

### Isolation of and culture conditions for murine and human HSPCs

Murine bone marrow cells were flushed from the femora and tibiae of donor mice. Erythrocytes were lysed. Murine Lin^−^ c-kit^+^ sca-1^+^ HSPCs were isolated by magnetic sorting (Indirect Lineage Cell Depletion Kit, Milteny Biotec containing lineage-specific antibodies directed against CD5, CD45R/B220, CD11b, GR-1/Ly-6G/C), 7-4, and Ter-119) [7]. CD34^+^ human HSPCs were isolated from umbilical cord blood of healthy male and female donors using the CD34 MicroBead Kit (Milteny Biotec) [7]. Murine and human HSPCs were maintained in cell culture as described [7]; details are also summarized in the supplementary information.

### Expression of DPP4/CD26

Murine and human HSPCs were pretreated with treprostinil (10 μM) and forskolin (30 μM) for 1 to 6 h at 37 °C (7). Untreated cells served as control. Subsequently, human cells were stained with the FITC-labeled 4H11-antibody against CD34 and the phycoerythrin-labeled 2A6-antibody against human CD26. CD34^+^ cells were gated to quantify the surface expression of human CD26 in a FACSCanto II (Becton-Dickinson). Murine cells were stained with the PerCP-Cyanine 5.5 H194-112-antibody against murine CD26. Surface expression was assessed by quantifying median fluorescence intensity (MFI) and normalized to control.

### Chemotaxis assay

Chemotaxis of murine and human HSPCs towards SDF-1/CXCL12 was determined using a two-chamber Transwell™ system. HSPCs were incubated in vitro in the absence and presence of 10 μM treprostinil and 30 μM forskolin for 1 h at 37 °C (7). Subsequently, the washed cell suspension (2 × 10^5^ in 0.1 ml) was added to the upper chamber. Medium supplemented with 100 ng ml^−1^ SDF-1/CXCL12 was added to the lower chamber. In some cases, vildagliptin (30 nM) was added to the upper and lower chamber during migration. After 4 h at 37 °C, the number of cells in the lower chamber was counted in a Luna automated cell counter (Logos Biosystems) and was expressed as percentage of the total cells originally added to the upper chamber.

### Colony formation

Murine bone marrow cells isolated from 6- to 8-week-old mice were resuspended in MethoCult™ GF M3434, which had been supplemented with either treprostinil (10 μM), vildagliptin (30 nM), or the combination thereof. The cell suspensions (2.5 × 10^4^ ml^−1^) were plated on 35 mm culture dishes and cultured at 37 °C in an atmosphere containing 5% CO_2_ for 11 days. The number of mixed colonies, which formed in the semi-solid methylcellulose, was counted under a light microscope using a scoring grid (5-fold magnification). Distinct colony types (CFU-GEMM, CFU-GM, and CFU-G) were assessed by their morphology.

### Transplantation of murine and human HSPCs

Homing and engraftment/bone marrow reconstitution by murine or human HSPCs was assessed by subjecting mice to HCT as previously described [7]. Homing of murine HSPCs was assessed by flow cytometry and by a colony-forming cell (CFC) assay. After 16 h, a suspension of bone marrow cells was obtained from femura and tibiae of recipient mice. Aliquots were either stained with an antibody directed against CD45.1 or resuspended in MethoCult™ containing granulocyte-macrophage colony-stimulating factor and IL-3 (10 ng ml^−1^ each) for the formation of granulo-monocytic colony-forming units and 3 IU ml^−1^ of erythropoietin and IL-3 for erythroid colony-forming units. The number of colonies formed within 11 days was counted under a light microscope.

## Results

### Surface expression of DPP4/CD26 on HSPCs is stimulated by treprostinil and forskolin

**Fig. 1:**
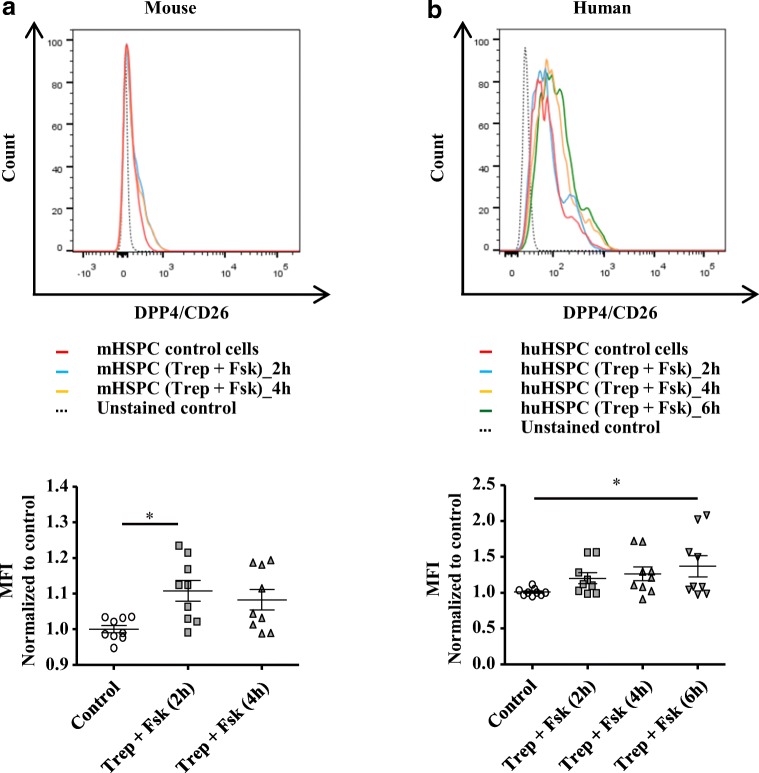
Surface levels of DPP4/CD26 on murine and human HSPCs stimulated by the combination of treprostinil and forskolin. **a** Murine lin^−^ cells and **b** human CD34+ HSPCs were incubated in the absence (red trace = vehicle control) or in the presence of 10 μM treprostinil and 30 μM forskolin for 2 (blue trace), 4 (yellow trace) or 6 h (green trace) at 37 °C and stained for CD26/DPP4. The upper panels show original flow cytometry histograms. Median fluorescence intensity (MFI) and respective values normalized to control are illustrated in bar graphs. Data are shown from *n* ≥ 3 independent experiments. Statistically significant differences were examined using Friedmann-test followed by Dunn’s multiple comparison test (*, *P* < 0.05)

DPP4/CD26 degrades SDF-1/CXCL12 [11]. DPP4/CD26 is expressed by human cord blood-derived CD34^+^ HSPCs and limits activation of CXCR4 by its cognate ligand SDF-1/CXCL12 [10]. Accordingly, we detected DPP4/CD26 on the surface of both, murine (Fig. 1a) and human HSPCs (Fig. 1b) by flow cytometry. The combination of treprostinil+forskolin increases expression of CXCR4 by HSPCs [7]. We surmised that this effect was balanced by a concomitant elevation of DPP4/CD26 surface levels. This was the case: incubation of murine HSPCs in the presence of treprostinil+forskolin led to a modest, but statistically significant increase of DPP4/CD26 on the surface (Fig. 1a). This increase was seen regardless of whether the cells were maintained in the continuous presence of treprostinil+forskolin (Fig. 1a) or only stimulated for 1 h and then incubated in fresh medium (data not shown). The response of human HSPCs was delayed, resulting in a statistically significant increase after 6 h (Fig. 1b).

### Migration and homing of HSPCs pretreated with treprostinil and forskolin is enhanced after blockage of CD26/DPP4 by vildagliptin

**Fig. 2:**
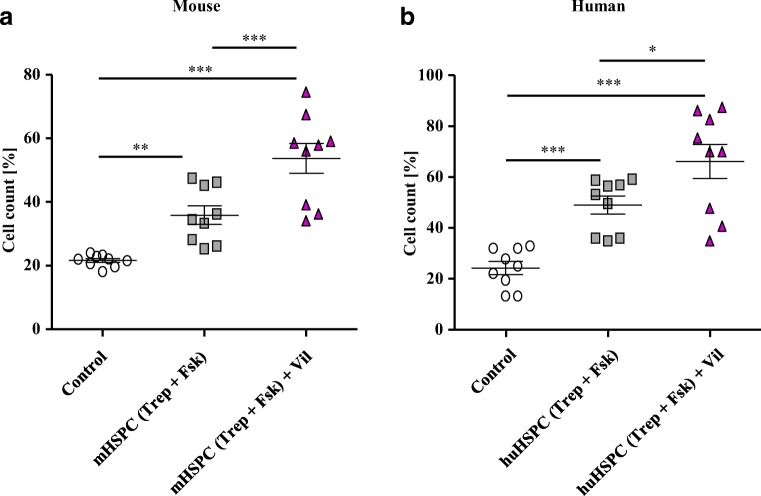
Pretreated murine and human HSPCs migrate more efficiently upon inhibition of CD26/DPP4 with vildagliptin. **a** Murine lin^−^ and **b** human CD34+ HPSC were pretreated in vitro with either vehicle or with the combination of 10 μM treprostinil and 30 μM forskolin for 1 h at 37 °C. 2 × 10^5^ murine or human HSPCs cells in 0.1 ml cell suspension was added to the upper chamber of a Transwell™ and allowed to migrate towards SDF-1 (100 ng ml^−1^) in the lower chamber for 4 h. In some samples, as indicated, 30 nM vildagliptin was added in to both chambers during incubation at 37 °C. Cells, which had migrated through the 5-μm filter, were counted via cell counter. Data are shown from three independent experiments. The statistical comparison was done by repeated measures ANOVA followed by Tukey’s multiple comparison (*, *P* < 0.05; **, *P* < 0. 01; *** *P* < 0.001)

The chemoattractant effect of SDF-1/CXCL12 is enhanced in HSPCs preincubated with treprostinil+forskolin [7]. This pretreatment also increased the surface levels of DPP4/CD26 indicating the presence of a negative feedback loop (Fig. 1). We explored, if inhibition of DPP4/CD26 rendered HSPCs more responsive to SDF-1/CXCL12-induced chemotaxis. Accordingly, after pretreatment of HSPCs with treprostinil+forskolin, vildagliptin (30 nM) was added during migration to block degradation of SDF-1/CXCL12. In the presence of vildagliptin, the number of both murine (Fig. 2a) and human HSPCs (Fig. 2b) in the lower chamber increased on average by 1.5-fold and 1.4-fold, respectively.

**Fig. 3:**
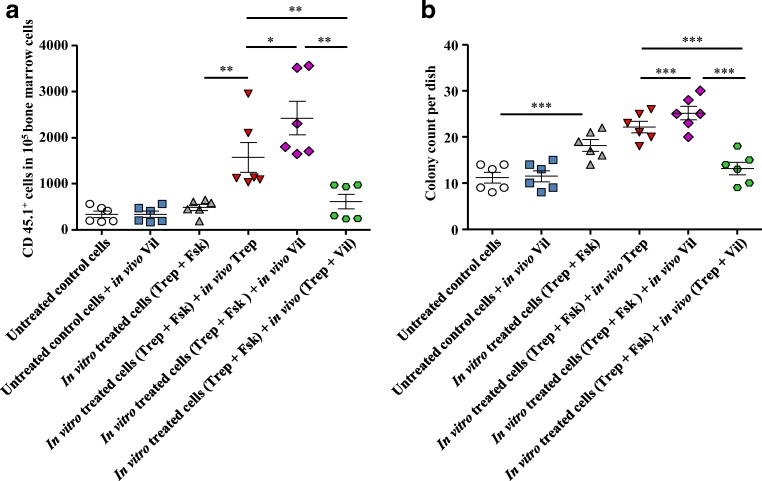
Treatment of recipient mice with vildagliptin increases homing of murine HSPCs. **a** and **b** CD45.1^+^ HSPCs isolated from bone marrow of B6.SJL-PtrcAPep3B/BoyJ donor mice were preincubated with either the combination of 10 μM treprostinil and 30 μM forskolin or vehicle control for 1 h at 37 °C. After washing, 2 × 10^5^ cells were injected into lethally irradiated (9.5 Gy) recipient C57Bl/6 (CD45.2^+^) mice. After transplantation, recipient mice were subcutaneously injected with either treprostinil (0.15 mg kg^−1^ 8 h^−1^), vildagliptin (30 mg kg^−1^ 24 h^−1^), or the combination thereof. Control mice received equal amounts of sterile water. Sixteen hours after transplantation, bone marrow cells were isolated from recipient mice. After lysis of erythrocytes, the cells were either stained for CD45.1 and CD45.2 to determine the proportion of CD45.1^+^ and CD45.2^+^ cells in the bone marrow by flow cytometry (**a**) or plated in MethoCult and incubated for 10 days to evaluate colony formation by the CD45.1^+^ donor cells, which had reached the recipient bone marrow (**b**). The statistical comparison was done by repeated measures ANOVA followed by Tukey’s multiple comparison test (*, *P* < 0.05; **, *P* < 0. 01; ***, *P* < 0.001)

The synergistic action resulting from stimulation of cAMP accumulation and inhibition of DPP4/CD26 is predicted to translate into enhanced homing of HSPCs in vivo. This prediction was verified by injecting murine HSPCs obtained from B6.SJL-PtrcAPep3B/BoyJ (CD45.1^+^) mice into lethally irradiated C57BL/6 (CD45.2^+^) recipient mice. Prior to transplantation, donor cells (CD45.1^+^) were preincubated in vitro for 1 h in the absence (untreated control) and presence of treprostinil+forskolin (in vitro treated cells). After transplantation, treprostinil (0.15 mg kg^−1^ 8 h^−1^), vildagliptin (30 mg kg^−1^), or the combination thereof was administered to recipient mice. Sixteen hours after transplantation, the bone marrow of recipient mice was analyzed for the presence of donor cells (CD45.1^+^) by flow cytometry. In vitro pretreatment with treprostinil+forskolin and subsequent in vivo administration of treprostinil increased homing of CD45.1^+^ cells by almost 5-fold (red triangles, Fig. 3a). However, when compared with the in vivo administration of treprostinil, the in vivo treatment with vildagliptin was more effective resulting in a 1.5-fold higher number of donor cells in the recipient bone marrow (cf. magenta and red triangles, Fig. 3a). In contrast, we did not detect an appreciable effect of vildagliptin on homing of untreated control HSPCs (blue squares, Fig. 3a). Surprisingly, concomitant administration of treprostinil and vildagliptin considerably reduced homing such that the number of donor cells was comparable to that of animals, which had been solely injected with HSPCs incubated in vitro with treprostinil+forskolin without any additional treatment in vivo (cf. green and gray symbols, Fig. 3a).

Thus, when administered concomitantly in vivo*,* treprostinil and vildagliptin had a mutually antagonistic effect on homing of HSCPs. We corroborated this surprising finding in a colony-forming cell (CFC) assay. This was used as an independent read-out, because it allowed for identifying hematopoietic progenitors, which had homed into the recipient bone marrow: in semi-solid methylcellulose supplemented with factors required for the formation of granulo-monocytic colony-forming units and for erythroid colony-forming units, these cells gave rise to colonies within 10 days. The results obtained by flow cytometry (Fig. 3a) were indeed reflected by the number of colonies formed (Fig. 3b).

### Regimen-dependent synergism or antagonism by vildagliptin in a murine model of HCT

**Fig. 4:**
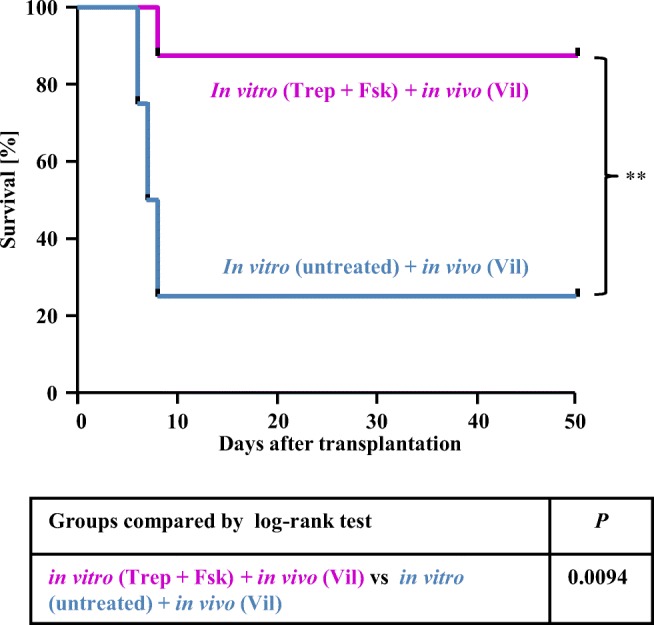
Vildagliptin enhances bone marrow reconstitution by murine HSPCs pre-treated with the combination of treprostinil and forskolin. Murine lin^−^ HSPCs were incubated in vitro with either vehicle (control) or 10 μM treprostinil and 30 μM forskolin for 1 h at 37 °C. A washed suspension of 2 × 10^5^ HSPCs was injected via the tail vein into lethally irradiated recipient BALB/c mice. Recipient mice indicated by the light blue curve and the magenta curve received untreated control cells (in vitro untreated; *n* = 8) and cells preincubated with treprostinil and forskolin (in vitro Trep + Fsk), respectively, and were subsequently administered vildagliptin (in vivo Vil) by subcutaneous injection (30 mg kg^−1^ 24 h^−1^, *n* = 8). The table summarizes the comparison of the survival curves by log-rank test (**, *P* < 0. 01)

The observations illustrated in Fig. 3 indicated that treatment of recipient mice with vildagliptin conferred an advantage to HSPCs, which underwent homing into the bone marrow provided that they had been preincubated in the presence of treprostinil+forskolin. We confirmed this finding by injecting murine HSPCs pretreated with treprostinil+forskolin into lethally irradiated recipient mice. These mice were subsequently administered vildagliptin (30 mg kg^−1^ 24 h^−1^ s.c.; magenta curve, Fig. 4). The survival of these mice was enhanced when compared with mice, which had been transplanted with untreated murine HSPCs and which subsequently received vildagliptin (blue curve, Fig. 4).

**Fig. 5:**
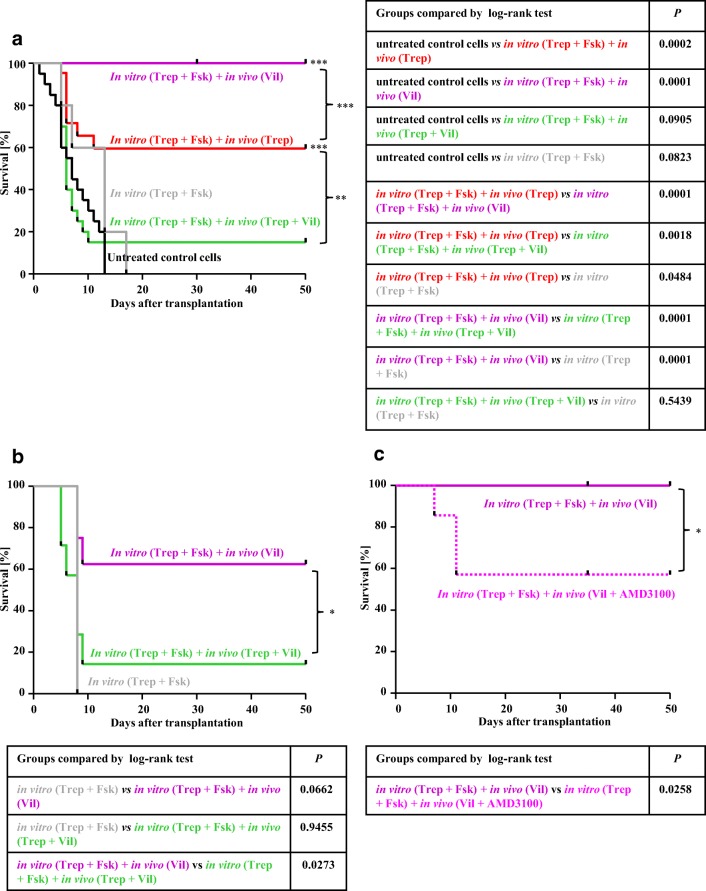
Sole administration of vildagliptin enhances bone marrow reconstitution in a murine (**a**, **c**) and a humanized mouse model (**b**) of HCT. **a** Murine HSPCs were incubated in vitro with either vehicle (control) or 10 μM treprostinil and 30 μM forskolin (Trep + Fsk) for 1 h at 37 °C. A washed suspension of 2 × 10^5^ HSPCs was injected via the tail vein into lethally irradiated recipient BALB/c mice. Recipient mice were divided into 5 groups: control mice received untreated cells (black curve, *n* = 20); mice represented by the gray curve were transplanted with HSPCs pretreated in vitro (in vitro Trep + Fsk, *n* = 6), those represented by the red curve were transplanted with in vitro pretreated cells and were administered treprostinil (in vivo Trep) by subcutaneous injection (0.15 mg kg^−1^ 8 h^−1^, *n* = 22); recipient mice indicated by the magenta curve were injected cells preincubated with treprostinil and forskolin (in vitro Trep + Fsk) and were subsequently administered vildagliptin (in vivo Vil; 30 mg kg^−1^ 24 h^−1^, *n* = 20); the group of recipient mice represented by the green curve (*n* = 20) were injected in vitro preincubated cells and were administered the combination of treprostinil and vildagliptin (in vivo Trep +Vil). **b** Treatment of NSG recipient mice with vildagliptin stimulates the engraftment of pretreated human HSPCs. Human CD34^+^ HSPCs were incubated in vitro with 10 μM treprostinil and 30 μM forskolin for 1 h at 37 °C. A washed suspension of 1.5 × 10^5^ human CD34^+^ HSPCs was injected via tail vein into lethally irradiated recipient NSG mice. Recipient mice indicated by the gray curve were transplanted with HSPCs pretreated in vitro (in vitro Trep + Fsk, *n* = 4), those represented by the magenta curve were subsequently administered vildagliptin (in vivo Vil) by subcutaneous injection (*n* = 8); recipient mice indicated by the green curve were administered the combination of vildagliptin and treprostinil (in vivo Vil + Trep; *n* = 7). **c** In vivo, the beneficial effect of vildagliptin is only partially abrogated by the CXCR4-antagonist plerixafor AMD3100. Murine HSPCs (2 × 10^5^ HSPCs/recipient) were incubated in vitro with 10 μM treprostinil and 30 μM forskolin and subsequently injected into lethally irradiated recipient BALB/c mice as outlined for panel **a**. Recipient mice represented by the magenta curve and the dotted pink curve were administered vildagliptin (30 mg kg^−1^ 24 h^−1^, *n* = 10) and vildagliptin in combination with AMD3100/plerixafor (3.3 mg kg^−1^ 8 h^−1^) by subcutaneous injection (*n* = 7). The tables summarize the statistical comparisons of the individual Kaplan-Meier plots by log-rank test (*, *P* < 0.05. **, *P* < 0. 01; ***, *P* < 0.001)

In contrast, the combined in vivo administration of treprostinil (0.15 mg kg^−1^ 8 h^−1^) and vildagliptin resulted in mutual antagonism in homing (Fig. 3). We verified the relevance of these findings for engraftment and reconstitution of the bone marrow: lethally irradiated mice were administered a limiting number of HSPCs (i.e., 2 × 10^5^ cells); recipient animals were not rescued, if these cells had not been pretreated prior to their injection (black curve, Fig. 5a). About 60% of the mice survived, if the HSPCs had been first incubated in the presence of the combination of treprostinil+forskolin and the animals were then administered treprostinil (red curve, Fig. 5a). However, the most effective regimen was to administer pretreated HSPCs followed by in vivo administration of vildagliptin, because all recipient animals survived (magenta curve, Fig. 5A). The outcome of the treprostinil- and the vildagliptin-treated group differed in a statistically significant manner (Fig. 5a). In contrast, if mice were subjected to a combined treatment with treprostinil and vildagliptin, their survival was substantially lower (green curve, Fig. 5a) than that seen after treatment with either agent. In fact, it did not differ significantly from that of animals, which had only been injected untreated HSPCs (black curve, Fig. 5a). Thus, when administered concomitantly, treprostinil and vildagliptin exerted a mutual antagonism, resulting in poor engraftment of HSPCs and subsequent reconstitution of hematopoiesis.

We verified that this mutual antagonism was also relevant for the human situation by injecting CD34^+^ human HPSCs isolated from umbilical cord blood into lethally irradiated NSG mice. The bone marrow niche of NSG mice is permissive to engraftment of human HSPCs [12]. We focused on two conditions to reveal regimen-dependent agonism and antagonism by vildagliptin. Recipient mice were injected CD34^+^ human HPSCs pretreated with treprostinil+forskolin and were then administered solely vildagliptin or the combination of treprostinil and vildagliptin by subcutaneous injection in vivo (magenta and green curve in Fig. 5b). The fate of recipient mice transplanted with pretreated human HSPCs was dependent on the presence of treprostinil in the subsequent in vivo treatment regimen: the survival of vildagliptin-treated recipient mice was reduced by concomitant administration of treprostinil (green curve, Fig. 5b). Thus, if administered concomitantly, treprostinil and vildagliptin exerted a mutual antagonism, which was also evident in a humanized mouse model of HCT.

### Concomitant administration of the CXCR4-antagonist plerixafor/AMD3100 and vildagliptin to recipient mice

The beneficial action of treprostinil on HSPCs engraftment is completely abrogated, if recipient mice are administered the CXCR4 antagonist plerixafor/AMD3100 [7]. We surmised that the improved survival observed in vildagliptin-treated mice was also accounted for by enhanced stimulation of CXCR4. Accordingly, we administered plerixafor/AMD3100 (3.3 mg kg^−1^ 8 h^−1^) together with vildagliptin for 10 days to recipient mice, which had been injected HSPCs pretreated with treprostinil/forskolin. Plerixafor/AMD3100 only reduced—but did not eliminate—the survival advantage conferred by vildagliptin. In fact, about 40% of the recipient mice survived in spite of the treatment with plerixafor (dotted pink curve, Fig. 5c). This observation suggests that vildagliptin enhances survival by a mechanism other than enhanced SDF-1-mediated stimulation of CXCR4.

### Enhanced colony formation by murine HSPCs in the presence of vildagliptin is reduced in the concomitant presence of treprostinil

**Fig. 6:**
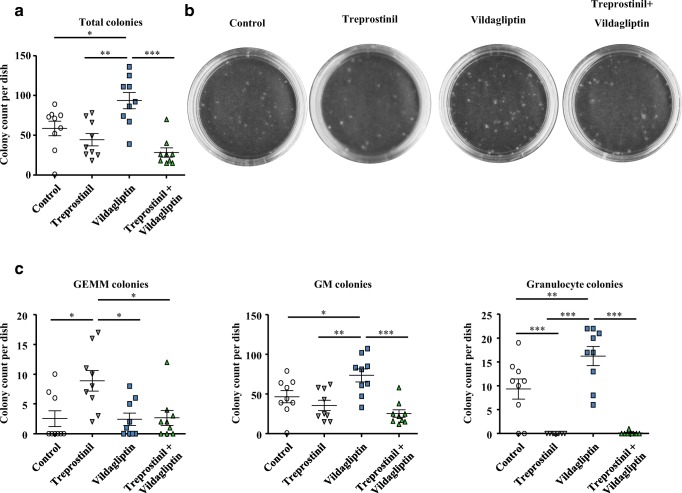
Stimulation by vildagliptin of colony formation by murine HSPCs. **a** Murine HSPCs were isolated form bone marrow, resuspended in a MethoCult™ GF M3434 and were plated on 35 mm culture dishes in the absence and presence of 10 μM treprostinil, 30 nM vildagliptin or the combination thereof. Dishes were cultured at 37 °C and 5% CO_2_ for 11 days. The number of mixed colonies was counted under a light microscope (× 5 magnification). Data are shown from three independent experiments. The statistical comparison was done by repeated measures ANOVA followed by Tukey’s multiple comparison (*, *P* < 0.05; **, *P* < 0. 01; ***, *P* < 0.001). **b** Shown are representative photographs of total colonies on a 35-mm culture dish. **c** Individual colonies were assessed by their morphology to quantify CFU-GEMM, CFU-GM, and CFU-G

Repopulation of the bone marrow also depends on the ability of HSPCs to proliferate and to differentiate. CD26 does not only cleave chemokines and gut peptides but also several hematopoietic growth factors, which harbor a cleavage site in their N-terminus, e.g., GM-CSF, G-CSF, erythropoietin, and IL-6 [13]. The resulting truncated growth factors act as antagonists [14]. In Fig. 6a and b we show the effect of individual agents on mixed colonies comprising all lineages, which hence represent the earliest progenitors and the stem cells: the number of mixed colonies, which emerged in semisolid methylcellulose, was increased, if murine HSPCs were maintained in the presence of 30 nM vildagliptin (blue symbols, Fig. 6a; 3rd photomicrograph, Fig. 6b). If HSPCs are preincubated for 1 h in the presence of treprostinil+forskolin, their subsequent proliferation and differentiation is not affected [7]. Here, we maintained murine HSPCs in the continuous presence of treprostinil: the total number of mixed colonies, which formed over 11 days, was slightly—albeit not significantly—lower (gray triangles, Fig. 6a). In contrast, if we assessed the effect of individual agents on distinct colony types (CFU-GEMM, CFU-GM, and CFU-G, Fig. 6c), treprostinil and vildagliptin enhanced the outgrowth of multi-lineage CFU-GEMMs (gray triangles, Fig. 6c, left hand panel) and of CFUs arising from the later stage committed progenitors (CFU-GM and CFU-G, green triangles, Fig. 6c, middle and right hand panel), respectively. However, when combined, treprostinil blunted the ability of vildagliptin to increase the number of both, total mixed colonies (green triangles, Fig. 6a; cf. 4th and 3rd photomicrograph in Fig. 6b) and GM- and G-colonies (green triangles, Fig. 6c, middle and right hand panel). Vice versa, in the combination, the enhancing effect of treprostinil on early progenitors was also abolished (cf. gray and green triangles, Fig. 6c, left hand panel). These observations highlight the mutual antagonism exerted by treprostinil and vildagliptin during hematopoietic differentiation.

## Discussion

Three G protein-coupled receptors have been shown to be involved in homing, lodging, and engraftment of HSCs, i.e., the receptors for E prostanoid [5–7] and Ca^2+^ [15, 16] and CXCR4 [17]. It is reasonable to assume that appropriate combinations of drugs, which target these receptors, can improve the outcome of HCT. In fact, the DPP4-inhibitor sitagliptin was previously shown to enhance engraftment of HSPCs, which had been preincubated in the presence of dimethylated PGE2 (dmPGE2) [9]. However, contrary to dmPGE2, which also activates G_i_-coupled E prostanoid receptors, treprostinil is selective for G_s_-coupled E and I prostanoid receptors, it has favorable pharmacokinetic properties, and it is approved for clinical use. Hence, treprostinil can be administered in vivo. Our experiments were designed to define the treatment regimen, under which treprostinil can be combined with DPP4-inhibtion by vildagliptin. We compared several regimen by injecting a limiting number of HSPCs: i.e., unless preincubated to raise cAMP prior to injection, the number of HSPCs did per se not suffice to rescue recipient animals [7]. The results were unequivocal: administration of vildagliptin allowed for survival of all lethally irradiated recipient mice provided that they had been injected HSPCs, which had been pretreated with trepostinil+forskolin in vitro (referred to as sequential regimen). Without this preincubation, vildagliptin only had a modest effect on survival. Sole injection of HSPCs pretreated with treprostinil+forskolin is effective in enhancing survival of recipient mice, but the additional treatment of recipients with treprostinil further enhances their survival and shifts the threshold of HSPCs to lower numbers [7]. However, here we showed in a head-to-head comparison that the vildagliptin-based regimen was superior to the treprostinil-based treatment in rescuing lethally irradiated recipient animals. The most surprising finding, though, was the mutual antagonism exerted by the combined administration of vildagliptin and treprostinil: recipient animals, which received both compounds, fared worse than recipients administered either compound. In fact, their survival was not different from those, which had been injected untreated HSPCs. Two independent observations confirmed this mutual antagonism: (i) administration of either vildagliptin or of treprostinil augmented the number of HSPCs, which were retrieved from the bone marrow of recipient mice 16 h after their injection. In contrast, the combined treatment—i.e., the concomitant administration of vildagliptin and of treprostinil—resulted in reduced homing of HSPCs, regardless of whether their number was assessed by staining for their unique surface marker CD45.1 or their ability to form colonies in methylcellulose. (ii) If lethally irradiated NSG mice were injected a limiting number of CB-derived human C34^+^ HSPCs, survival of vildagliptin-treated recipient animals was superior to that observed after administration of vildagliptin and treprostinil. This observation indicates that mutual antagonism is of relevance to human HCT.

The synergism observed in the sequential regimen—regardless of whether achieved with dmPGE2 and sitagliptin [9] or with treprostinil+forskolin and vildagliptin—can be rationalized: prostanoid receptor activation increases the expression of CXCR4 [7, 18]. Here we show that the preincubation with treprostinil also modestly increased the levels of DPP4/CD26 on the surface of HSPCs. Thus, CXCR4 upregulation was accompanied by increased deactivation of its cognate ligand SDF-1/CXCL12 indicative of a negative feedback loop [14]. Accordingly, inhibition of DPP4/CD26 is predicted to enhance the prior action of treprostinil: this prediction was verified by examining the ability of vildagliptin to enhance (i) migration of HSPCs towards SDF-1/CXCL12, (ii) homing of HSPCs, and (iii) reconstitution of hematopoiesis and thus survival of recipient mice. It is, however, evident that, in vivo, the action of vildagliptin cannot be solely accounted for by inhibition of SDF-1/CXCL12 degradation. The concomitant administration of the CXCR4-antagonist plerixafor/AMD3100 to recipient mice did not abolish the beneficial effect of vildagliptin on survival: about 40% of the recipient mice survived. In contrast, plerixafor/AMD3100 completely abrogated the survival enhancing action of treprostinil [7]. Inhibition of DPP4/CD26 also enhances the action of hematopoietic growth factors [14, 19]: this is likely to account for the increased number of colonies formed by HSPCs in the presence of vildagliptin. Importantly, treprostinil, which per se did not affect colony formation, antagonized the action of vildagliptin. It is tempting to speculate that this effect contributed to the mutual antagonism, which was seen when vildagliptin and treprostinil were administered concomitantly to recipient mice during bone marrow reconstitution. The mechanistic basis for this mutual antagonism remains enigmatic, but it may result from incongruent signals arising from the simultaneous stimulation of two or more pathways, which support synergism when activated sequentially. More importantly, these observations have translational implications: they strongly argue against the design of clinical trials, where concomitant administration of treprostinil and a DPP4-inhibitor is tested. They also highlight the importance of exploring any drug combination first in preclinical models of HCT.

Two phase I/II trials were conducted to explore the safety and efficacy of sitagliptin in cord blood transplantation [20, 21]: the first trial examined a dose of 600 mg sitagliptin/day. The observations suggested that engraftment and reconstitution of hematopoiesis was related to the extent of DPP4-inhibition [20]. Based on pharmacokinetic/pharmacodynamic modeling [22], a dose of 1200 mg/day was selected for the second trial [21]. Of note, these dosages are 6- and 12-fold higher than those administered in type-II diabetes. Nevertheless, the efficacy of 600 mg/day sitagliptin was modest [20]. Here, we employed 30 mg/kg/day, which is reasonably comparable—when corrected for allometry [23]—to the human dose in type-II diabetes. This dose of vildagliptin only rescued 30% of the recipient animals, which had received murine HSPCs, but sufficed to rescue ≥ 90% of the recipients transplanted with murine HSPCs preincubated with treprostinil+forskolin. Based on these findings, we posit that it is worthwhile considering clinical trials, which explore the synergism between cAMP-raising agents and standard doses of DPP4-inhibitors in a sequential regimen. This may be more beneficial than exposing patients to very high doses of DPP4-inhibitors, where off-target effects are likely to occur.

HCT can be curative for patients with hematological malignancies or genetic disorders. However, HSPCs also have immunoregulatory properties. In fact, transplantation of autologous HSPCs (AHSCT) has been proposed for the treatment of autoimmune diseases [24, 25]. Apart from CXCR4 [26], it is the modulation of PD-L1 expression, which represents an attractive target for enhancing immunoregulation by HSPCs [24, 25]. Interestingly, PGE2-analogs increase the expression of CXCR4 and PD-L1 [27]. Hence, it is attractive to explore, if treprostinil can be repurposed to facilitate AHSCT.

## Electronic supplementary material

ESM 1 (PDF 127 kb)

## Abbreviations

AMD3100: Plerixafor
cAMP: Cyclic adenosine monophosphate
CaSR: Calcium-sensing receptor
CB: Cord blood
CFC: Colony-forming cell
CSF: Colony-stimulating factor
CXCR4: C-X-C motif chemokine receptor 4
DPP4: Dipeptidylpeptidase-4
Fsk: Forskolin
HCT: Hematopoetic cell transplantation
HSCs: Hematopoietic stem cells
HSPCs: Hematopoietic stem and progenitor cells
SDF-1: Stromal cell-derived factor-1
Trep: Treprostinil
Vil: Vildagliptin
