# Supplementary material for: Regimen-dependent synergism and antagonism of treprostinil and vildagliptin in hematopoietic cell transplantation
Source: J Mol Med (Berl). 2019 Dec 24;98(2):233–43. doi: 10.1007/s00109-019-01869-8 (PMC7007891; doi:10.1007/s00109-019-01869-8)
Supplement: Supplementary file 1 — (PDF 127 kb) [file 109_2019_1869_MOESM1_ESM.pdf]

Regimen-dependent Synergism and Antagonism of Treprostinil and Vildagliptin in Hematopoietic Cell Transplantation

Supplemental Materials and Methods

Materials

Treprostinil was provided by SciPharm SàRL (L-2540 Luxembourg City, Luxembourg); vildagliptin was purchased from Cayman Chemical (Ann Arbor, MI). StemSpan serum-free expansion medium (SFEM), EasySep Human Cord Blood CD34 Positive Selection Kit, and MethoCult™ GF M3434 [1] for murine HSPCs were purchased from STEMCELL Technologies (Vancouver, BC, Canada); Lineage Cell Depletion Kit was obtained from Miltenyi Biotec GmbH (Bergisch Gladbach, Germany). Growth factors and cytokines required for cell culture were from PeproTech (Vienna, Austria). Chemicals for flow cytometry were from BD Biosciences (Schwechat, Austria). Antibodies against human CD34 (4H11; FITC-labeled), human CD26 (2A6; phycoerythrin-labeled), murine CD26 (H194-112; PerCP-Cyanine 5.5-labeled), murine CD45.1 (A20; phycoerythrin-labeled), and murine CD45.2 (104; FITC-labeled) were from eBioscience (San Diego, CA); recombinant human stromal cell-derived factor-1 (rhSDF-1), and recombinant murine SDF-1 were purchased from R&D Systems, Inc. (Minneapolis, MN). AMD3100 was purchased from Abcam (Cambridge, UK) and all other chemicals from Sigma-Aldrich (St. Louis, MO). Two-chamber-Transwells (6.5-mm diameter, 5.0 µm pore size) was obtained from Corning Life Sciences (Tewksbury MA).

Isolation of murine and human hematopoetic stem and progenitor cells

The long bones of the hind limbs (*i.e.*, femora and tibiae) of donor mice were freed of muscle and connective tissue and flushed with RPMI medium using a syringe and 27½ G needle. The cell suspension was freed from visible connective tissue, collected and transferred to

centrifuge tubes. Cells were harvested by centrifugation (1,200 rpm/~100 g for 5 min) and resuspended in 3 mL erythrocyte lysis buffer (0.15 M NH_4Cl, 10 mM $KHCO_3$, 0.1 mM EDTA, pH adjusted to 7.2 to 7.4). The cell suspension was incubated for 2 min at 20°C followed by 4 min on ice. Thereafter, RPMI (10 mL) was added and the cells were harvested by centrifugation and counted. The typical yield of cells was $3*10^7$/mouse.

Cells were resuspended in ice-cold PBS (phosphate buffered saline) containing 2% FCS (fetal calf serum) at a cell density of $2.5 * 10^8$ cells/mL to which a cocktail of biotinylated antibodies ("Lineage Cell Depletion Kit" of Miltenyi Biotec) containing lineage-specific antibodies directed against CD5, CD45R (B220), CD11b, GR-1 (=Ly-6G/C), 7-4, and Ter-119 at a ratio of 0.1 mL antibody solution per 10^8 cells. Cells were incubated for 20 min on ice with the antibodies and pelleted by centrifugation. After resuspension ($3.3 *10^8$ cells/mL), the second anti-biotin-coated MicroBeads (0.2 mL/10^8 cells, provided with the ("Lineage Cell Depletion Kit" of Miltenyi Biotec) was added to the cell suspension and the mixture was incubated for 15 min on ice. Thereafter, the sample was diluted in MACS-buffer (30 mL), the cells were collected by centrifugation and resuspended in 6 mL of MACS-buffer. This suspension was loaded onto prepacked LS columns, which contain ferromagnetic beads coated with a cell-compatible plastic material. Typically three columns were employed for (2 mL cells suspension/column). The flow-through contained the lineage marker-negative cells (lin^- cells), while the lineage committed cells were retained on the column. Cells were pelleted by centrifugation and resuspended in 2 mL PBS. The typical yield was $7*10^5$ lin^- cells/mouse.

Human hematopoetic stem and progenitor cells (HSPCs) were harvested from umbilical cords of healthy donors: cord blood samples (≅50 mL) were collected during healthy full-term deliveries. CD34+ cells were isolated using magnetic-activated cell-sorter (MACS) Direct CD34 Progenitor Cell Isolation Kit (Miltenyi Biotech) and expanded as described [2]. Briefly, cord blood was diluted with an equal volume of phosphate-buffered saline (PBS); this

suspension (25 ml) was layered onto LymphoPrep™ (a density medium obtained through Nycomed which contains a mixture of sodium triatoate and polysaccharides). The tubes were centrifuged in a swinging bucket rotor for 30 min at 355 g. The layer containing mononuclear cells was harvested, diluted with PBS (to 50 mL) and centrifuged at 400 g for 8 min to remove residual LymphoPrep™. Erythrocytes were removed by lysis in buffer containing 150 mM NH_4Cl, 10 mM $KHCO_3$ and 0.1 mM EDTA (pH adjusted to 7.2 to 7.4 with HCl) for 10 min at 4°C. The number of mononuclear cells was determined and adjusted to $2*10^8$ cells/mL in the MACS buffer provided with the Isolation Kit. The EasySep® Positive Selection Cocktail was added (0.1 mL/mL cell suspension), the suspension was incubated for 15 min at room temperature and EasySep® Magnetic Nanoparticles (50 µL/mL) were added. After an additional incubation for 10 min at room temperature, the cell suspension was diluted to 2.5 mL by the addition of medium. The tube was placed into the magnet for 5 min and cells subsequently collected. This step was repeated 5 times. The enriched cells were propagated for 6 days (*i.e.*, two population doublings) in suspension cultures containing serum-free X-VIVO15 medium (BioWhittaker) supplemented with GlutaMAX (2.5 mM; Gibco/Invitrogen) and penicillin/streptomycin (P/S; 125 U/mL each) and Flt3L, SCF and TPO (each at 50 ng/mL). Typical yields were $9*10^5$ CD34+ cells/cord blood specimen, which were expanded to obtain $3.5 * 10^6$ cells. All procedures were carried out in accordance to the guidelines from the Medical University of Vienna Institutional Review Board for these studies. Informed consent was provided in accordance with the Declaration of Helsinki Principles.

Transplantation of Murine and Human HSPCs

All recipient mice were identified by numbered ear tags. These numbers were randomly allocated to treatment groups.

a) Homing of murine HSPCs was assessed by flow cytometry and by a colony forming cell (CFC) assay. Regardless of the read out system, murine HSPCs were isolated from the bone marrow of $CD45.1^+$ B6.SJL-PtrcAPep3B/BoyJ donor mice. Murine HSPCs were incubated in the presence of treprostinil (10 μM) and forskolin (30 μM) or vehicle control. After pre-treatment, $2*10^5$ $CD45.1^+$ HSPCs were injected into the lethally irradiated (9.5 Gy, split doses, 2 Gy min^{-1}; Siemens Primus, 6MV, Siemens Austria) $CD45.2^+$ C57BL/6J recipient mice. After transplantation, recipient mice were subcutaneously injected with either treprostinil (0.15 mg kg^{-1} 8 h^{-1}), vildagliptin (30 mg kg^{-1} 24 h^{-1}) or with the combination thereof. Untreated control mice received sham injections of 0.1 ml.

After 16 hours, the whole bone marrow obtained from femura and tibiae of recipient mice was analyzed by flow cytometry for expression of CD45.1 and CD45.2 on their surface or seeded in MethoCult™ to evaluate colony formation. Cultures were started with total mono-nuclear bone marrow cells resuspended in MethoCult containing granulocyte-macrophage colony-stimulating factor and IL-3 (10 ng ml^1 each) for the formation of granulo-monocytic colony–forming units and 3 IU ml^1 of erythropoietin and IL-3 for erythroid colony forming units and cultured at 37°C and 5% CO2 for 10 days. The number of total colonies which formed within 10 days was counted under a light microscope.

b) Engraftment and bone marrow reconstitution by murine or human HSPCs was assessed by subjecting mice to HCT as previously described [1]: in brief, recipient BALB/c or NSG mice underwent lethal irradiation (9 Gy, split doses, 2 Gy min^{-1}; Siemens Primus, 6MV, Siemens Austria). After lethal irradiation, mice were allowed to recover for 24 hours. Prior to transplantation, murine HSPCs from BALB/c donor mice or human umbilical cord blood-derived HSPCs were pre-incubated in the absence and presence of 10 μM treprostinil and 30 μM forskolin for 1 hour at 37°C. The incubation medium was subsequently removed by three cycles of washing to avoid any carryover effects. Via tail vein injection, murine and human

HSPCs (pretreated or control donor cells) were administered into BALB/c or NSG recipient mice. Limiting numbers of cells were transplanted as verified previously [2]: $2*10^5$ murine HSPCs were transplanted in BALB/c mice illustrated in Fig 4, 5A and 6A and $1.5*10^5$ human HSPCs in NSG mice depicted in Fig. 5B. The transplantation was performed by a second person, who was blinded to the syringe content. Recipient mice were also subjected to an *in vivo* treatment. Starting immediately prior to transplantation of cells, recipient mice were injected subcutaneously with treprostinil (0.15 mg kg^{-1} 8 h^{-1}), vildagliptin (30 mg kg^{-1} 24 h^{-1}), the combination of treprostinil (0.15 mg kg^{-1} 8 h^{-1}) and vildagliptin (30 mg kg^{-1} 24 h^{-1}) or with the combination of vildagliptin (30 mg kg^{-1} 24 h^{-1}) and AMD3100 (3.3 mg kg^{-1} 8 h^{-1}) in a total volume of 0.1 ml per mouse, for 10 days. Untreated control mice received sham injections of 0.1 ml. The person who performed the injections *in vivo* was blinded to the syringe content. The animal technicians, who were blinded to the treatment, examined the wellbeing of the animals on a daily basis. They assessed whether a humane endpoint had been reached, which included emaciation (i.e., weight loss >30%), loss of activity, loss of grooming fur, or labored breathing. Mice meeting these criteria were sacrificed by cervical dislocation. If not rescued by transplantation of HSPCs, lethally irradiated mice succumbed to death within the first 2 weeks.

Supplemental References

1. Adelman DM, Simon MC (2002) Hypoxic gene regulation in differentiating ES cells. Methods Mol Biol 185:55-62

2. Kazemi Z, Bergmayr C, Prchal-Murphy M, Javaheri T, Themanns M, Pham HT, Strohmaier W, Sexl V, Freissmuth M, Zebedin-Brandl E (2016) Repurposing treprostinil for enhancing hematopoietic progenitor cell transplantation. Molr Pharmacol 89 (6):630-644.

3.
